# Pig farmers’ willingness to pay for management strategies to reduce aggression between pigs

**DOI:** 10.1371/journal.pone.0224924

**Published:** 2019-11-08

**Authors:** Rachel S. E. Peden, Faical Akaichi, Irene Camerlink, Laura A. Boyle, Simon P. Turner

**Affiliations:** 1 Animal Behaviour & Welfare, Animal and Veterinary Sciences Research Group, Scotland’s Rural College, Edinburgh, Scotland, United Kingdom; 2 Department of Rural Economy, Environment and Society, Scotland’s Rural College, Edinburgh, Scotland, United Kingdom; 3 Institute of Animal Welfare Science, University of Veterinary Medicine, Vienna, Austria; 4 Teagasc, Pig Development Department, Animal & Grassland Research and Innovation Centre, Fermoy, Co. Cork, Ireland; Universidade do Porto Instituto de Biologia Molecular e Celular, PORTUGAL

## Abstract

When deciding whether to invest in an improvement to animal welfare, farmers must trade-off the relative costs and benefits. Despite the existence of effective solutions to many animal welfare issues, farmers’ willingness to pay for them is largely unknown. This study modelled pig farmers’ decisions to improve animal welfare using a discrete choice experiment focused on alleviating aggression between growing/finishing pigs at regrouping. Eighty-two UK and Irish pig farm owners and managers were asked to choose between hypothetical aggression control strategies described in terms of four attributes; installation cost, on-going cost, impact on skin lesions from aggression and impact on growth rate. If they did not like any of the strategies they could opt to keep their current farm practice. Systematic variations in product attributes allowed farmers’ preferences and willingness to pay to be estimated and latent class modelling accounted for heterogeneity in responses. The overall willingness to pay to reduce lesions was low at £0.06 per pig place (installation cost) and £0.01 per pig produced (running cost) for each 1% reduction in lesions. Results revealed three independent classes of farmers. Farmers in Class 1 were unlikely to regroup unfamiliar growing/finishing pigs, and thus were unwilling to adopt measures to reduce aggression at regrouping. Farmers in Classes 2 and 3 were willing to adopt measures providing certain pre-conditions were met. Farmers in Class 2 were motivated mainly by business goals, whilst farmers in Class 3 were motivated by both business and animal welfare goals, and were willing to pay the most to reduce aggression; £0.11 per pig place and £0.03 per pig produced for each 1% reduction in lesions. Farmers should not be considered a homogeneous group regarding the adoption of animal welfare innovations. Instead, campaigns should be targeted at subgroups according to their independent preferences and willingness to pay.

## Introduction

Several animal welfare issues persist agriculture despite solutions being identified by extensive research (e.g. [1, 2]). Farm animals are business capital whose value is in direct proportion to their productivity, and although ensuring certain animal welfare standards is necessary to sustain productivity (e.g. feed, housing, disease control, environmental management), providing some improvements to animal welfare can be more expensive than the returns gained [3–5]. Therefore, it can be cheaper for farmers to accept losses due to certain welfare threats than to prevent these losses and, when deciding whether or not to invest in an animal welfare innovation, farmers must estimate and then trade-off the relative costs and benefits of making the change. Farmers are generally risk adverse, and in order to make an investment in animal welfare they must be confident that the costs will be covered by returns in productivity [6–12]. Nevertheless, farmers cannot be considered a homogeneous group. Their businesses differ in size, structure, location and management practices, farmers differ in personal attitudes and perceptions, and all of these factors can influence decisions with regards to animal welfare [9, 13–15]. Therefore, it is likely that farmers’ preferences and willingness to pay for animal welfare improvements are also heterogeneous. Although willingness to pay and its heterogeneity within a farming population are key determinants of whether effort will be made to improve animal welfare, these have rarely been estimated. This contrasts with the considerable research effort on identifying and refining management innovations to improve animal welfare. There is a need to bridge the gap between research and practice to facilitate the uptake of welfare solutions that fit within farmers’ willingness to pay constraints. Therefore it is crucial to: 1) understand how farmers make business decisions regarding specific animal welfare issues, and 2) account for heterogeneity in their preferences. In doing so, subgroups of farmers likely to invest in animal welfare can be identified, and campaigns to bridge the gap between research and practice can be targeted more effectively at farmers.

Aggression between pigs is one example of an animal welfare issue which has received extensive research, but seen inadequate progress in commercial practice [2]. Aggression occurs primarily when unfamiliar pigs are regrouped, as this disrupts their dominance hierarchy and they fight in order to re-establish dominance relationships [16]. Two surveys of 167 and 122 UK and Irish pig farmers found that farmers regroup unfamiliar pigs up to four times during each production cycle in order to maximise use of space, and in an attempt to create groups of equal body size which market simultaneously [17, 18]. Aggression threatens pig productivity as animals become more susceptible to infection due to the transient effects of stress upon the immune system [19], they acquire skin injuries as a result of bites [16, 20], and they are more likely to display lameness [21], slowed growth rates [22, 23] and reduced meat quality [24]. Several strategies to reduce the occurrence or intensity of aggression at regrouping have been identified, requiring farmers to make specific changes to animal management, nutrition or genetics (review articles: [25–27]). Some promising developments for use with growing/finishing pigs include housing pigs in relatively large social groups [28, 29], providing sufficient space at regrouping [30, 31], and providing a diet high in the amino acid tryptophan [32, 33]. Strategies are associated with monetary costs, and the extent and nature of these costs vary between strategies and between farms. Initial investment costs rely on business capital, and may result from the purchasing and fitting of new materials, or the restructuring or building of accommodation. Running costs may result from increased labour requirements and the replenishment of materials, and therefore mainly affect variable costs. Implementation of a strategy in practice may require only investment costs, only running costs, or a combination of both.

A recent survey found that although 88.5% of 122 UK and Irish pig farmers had tried an aggression control strategy in the past, only 41% expressed willingness to implement strategies again in the future [17]. Farmers’ willingness to implement strategies was influenced by a range of interrelated internal and external factors. Specifically these were: 1) their perception of their own ability to make the necessary management changes, 2) their beliefs about the likely outcomes, 3) their perception of aggression as a problem, and 4) their views of relevent stakeholder groups. Therefore, the limited uptake of aggression research in practice is linked to multiple factors. One of these is a potential misalignment between research and industry interests resulting in impractical or unaffordable solutions [17, 25]. No research to date has provided quantitative information on how farmers’ trade-off the relative costs and benefits of aggression control strategies when making investment decisions. Therefore, it is currently unknown whether any aggression control strategies result in benefits which are perceived to outweigh their associated costs. To encourage uptake of welfare solutions it is essential to better align research objectives with industry interests by identifying economically viable solutions based on farmers’ demands.

This research examines for the first time farmers’ preferences and willingness to pay (**WTP**) for reductions in aggression through a discrete choice experiment (**DCE**), and importantly, heterogeneity in farmer preferences is accounted for. DCEs are regularly used in a variety of research areas such as environmental valuation [34], transport systems [35], marketing [36] and healthcare economics [37, 38], and are generally accepted as a valid method for preference elicitation. DCEs are typically implemented in surveys and respondents are asked to indicate their preferred option among a number of different hypothetical alternatives with assigned prices. Preferences are measured via systematic variations of product characteristics (‘attributes’) in an experimental design. Based on respondent choices it is possible to estimate the relative importance of the different attributes [39, 40]. DCEs can also be used to estimate WTP for both changes in individual attributes, and changes in any combination of attributes [37, 38]. Furthermore, heterogeneity in these preferences can be understood by employing latent class modelling (**LCM**) [41] which identifies different classes of respondents based on both their demographic characteristics, attitudes and their preferences.

Specifically, this study aimed: 1) to measure farmers’ preferences for animal welfare improvements (i.e. reduction in lesions) and business goals (i.e. improvement in growth rate) when deciding to invest in an aggression control strategy, 2) to estimate farmers’ WTP for reductions in lesions and improvements in growth rate, and 3) to evaluate heterogeneity in their preferences and WTP. By identifying which attributes of aggression control strategies are attractive and important to farmers, and how they trade-off different costs and benefits, it may be possible to identify economically viable solutions that are compatible with these constraints. Furthermore, future research can be directed towards identifying new solutions which meet farmers’ demands. Crucially, by accounting for heterogeneity in these preferences, campaigns can be tailored and targeted at farmer subgroups according to their preferences and characteristics.

## Methods

### Experimental design

Farmers completed a paper based survey consisting of two sections. The first involved a choice task. Then, they were required to complete a questionnaire about their attitudes, farm characteristics and socio-demographic characteristics. Although previous research has successfully conducted choice experiments electronically, we chose to recruit participants face-to-face with a paper-based survey in order to maintain quality of responses and due to poor response rates of farmers to previous online surveys [17, 42]. Details of the sample size and recruitment process are given below in the section titled ‘Data collection’.

#### The choice task

In the choice task, farmers were asked to choose between four options. These options consisted of three hypothetical aggression control strategies (named simply as ‘strategy 1’ to ‘strategy 3’) which would be employed when regrouping unfamiliar growing/finishing pigs on their farm. As the use of aggression control strategies in practice is optional, it would be unrealistic to force farmers to choose a strategy. Therefore, we also included a status quo option to allow farmers to keep their current practice with regards to aggression and make no additional investment; hence mimicking the real life situation. Although pigs are more commonly mixed at weaning than at growing/ finishing [17, 18], the choice task focussed only on the growing and finishing stages of production. This is because farmers perceive aggression as a greater problem at growing/finishing compared to at weaning, and would be more likely to adopt a solution for these pigs [18]. Furthermore, at weaning, regrouping aggression has less impact on growth performance and less risk of lameness and injuries than at the grower/finisher stage when pigs are heavier and stronger [43]. Each alternative aggression control strategy was described in terms of four attributes: 1) One-off investment cost for installation of equipment or materials. As this cost would last for many production cycles, it was described ‘per pig place’; 2) Running cost associated with the running and management of the investment. As this cost would be required for every production cycle, it was described ‘per pig produced’; 3) Percentage reduction in lesions in the seven days following mixing (indicating a reduction in aggression [20]) and; 4) Percentage improvement in growth rate in the seven days following mixing achieved at the same level of food intake. Attributes 1 and 2 were expressed in Great British Pound Sterling and attributes 3 and 4 represented the change in lesions or growth rate relative to the farmer’s current practice. The levels for attributes 1 and 2 were assigned based on reviewing industry websites for products and relevant materials. The levels for attributes 3 and 4 were assigned based on the range found in the academic literature [16, 44–47]. Each attribute level was assigned an attribute level code as is required to design the level combinations (or ‘choice sets’, described below). See Table 1 for levels of each attribute and their associated codes.

**Table 1:** Attributes, attribute levels and attribute level codes for the hypothetical aggression control strategies.

Attribute	Attribute level	Attribute level code
One-off investment cost per pig place (£)	£1.22 per pig place	0
	£3.62 per pig place	1
	£6.12 per pig place	2
	£9.12 per pig place	3
Running cost per pig produced (£)	£0.12 per pig produced	0
	£0.24 per pig produced	1
	£0.34 per pig produced	2
	£0.46 per pig produced	3
Reduction in lesions (%)	10% reduction	0
	20% reduction	1
	30% reduction	2
	40% reduction	3
Improvement in growth rate (%)	0%	0
	2%	1
	5%	2
	8%	3

Given all the attribute levels, a full factorial design of 256 (i.e. 4x4x4x4) was created. Presenting the participants with 256 choices would be too time-consuming and demanding. Therefore, an optimal fractional factorial design was generated and used instead, whereby only a subset of the level combinations (or ‘choice sets’) appeared (see Table 2 for an example of a choice set). We followed the approach proposed by Street and Burgess (2007) [48] to generate an orthogonal design using the statistical software SPSS v17. A design is orthogonal if each attribute level appears an equal number of times, and the effects of each attribute on the participants’ responses can be independently estimated. The size of the orthogonal design (in this case 16 combinations) is equal to the minimum number of combinations required to meet these requirements. Prior research indicates that participants are able to complete 17 choice sets without problem [49]; therefore we saw 16 as a realistic number of choices for farmers to complete. These 16 combinations of the four attributes were used as the first aggression control strategy in each of the 16 choice sets (the ‘starting design’). Since participants were provided with choice sets of three aggression control strategies each plus a status quo option, the second and the third aggression control strategies were obtained using the generators (1111) and (2222), respectively. Generators were applied to the attribute level codes (see Table 1) to shift the levels of the attributes. See Street and Burgess (2007) for more details on how to obtain the generators and use them to generate the remaining options in a choice set.

**Table 2:** An example of a choice set presented to farmers.

Attributes	Unnamed strategy 1	Unnamed strategy 2	Unnamed strategy 3	Status quo
One-off investment cost per pig place (£)	£6.12	£9.12	£1.22	£0.00
Running cost per pig produced (£)	£0.34	£0.46	£0.12	£0.00
Reduction in lesions (%) during 7 days after mixing	10%	20%	30%	0%
Improvement in growth rate (%) during 7 days after mixing	5%	8%	0%	0%
Please mark the option you are most likely to adopt				

After generating all of the choice sets, each one of them was examined for implausible attribute combinations (e.g. where both investment and on-going costs were £0.00 but benefits to aggression and/or growth were positive), and attribute combinations that were clearly dominant (e.g. minimal costs and maximum benefits). These were removed to ensure a valid estimation process and to facilitate the perceived credibility of the tasks among the respondents, and the fractional factorial design was re-generated. Efficiency is a measure of the level of precision in which effects are estimated [34] and the final design had 100% statistical efficiency.

Hypothetical bias refers to bias caused when stated hypothetical WTP is higher than the real WTP, and is a major challenge for stated preference methods [50, 51]. To minimise the effect of hypothetical bias, first, a ‘cheap talk’ script was included to explicitly encourage participants to reveal their true preferences by answering exactly as they would if they had to choose an aggression control strategy for their farm, and had to pay for their choice. Second, an ‘opt-out reminder’ was used prior to each choice set. The opt-out reminder stated: ‘please remember that if you would not adopt any of the first three aggression control strategies on your farm, you should choose the ‘status quo’ option’. Hypothetical bias has been reduced by employing cheap talk scripts [51–53] and opt-out reminders [50] in prior research. Order effects were minimised by printing the choice sets in a different order for every respondent.

#### Attitudes, farm characteristics and socio-demographic characteristics

After successively completing the choice task, farmers who chose the ‘status quo’ for all the choice sets were asked to indicate why by ticking all relevant options from: ‘I would not adopt any of the aggression control strategies on my farm’, ‘I already use aggression control strategies on my farm’, ‘I don’t mix unfamiliar growers/ finishers’, ‘I didn’t read the other options’, ‘I wanted to get finished quickly’, and ‘Other…please state…’. All farmers were then asked to indicate their level of agreement/ disagreement (from 1 = strongly disagree to 5 = strongly agree) with seven attitudinal statements, specifically; 1) ‘when mixing unfamiliar pigs, minimizing aggression is important to me’, 2) ‘mixing aggression is a problem on my farm’, 3) ‘I avoid mixing unfamiliar pigs wherever possible’, 4) ‘it is possible to control aggression at mixing’, 5) ‘UK standards of pig welfare are sufficiently strict’, 6) ‘the welfare of my animals is important to me’, 7) ‘the welfare of my animals is good’. All farmers were also asked to indicate their use of the following eight aggression control strategies when mixing unfamiliar growing/finishing pigs on their farm by circling either ‘currently use’, ‘used in the past’ or ‘never used’: 1) ‘large social group sizes’, 2) ‘increased space allowance’, 3) ‘adding extra tryptophan to the feed’, 4) ‘solid visual barriers/ escape areas’, 5) ‘mixed weight groups’, 6) ‘mixing at night/ low light levels’, 7) ‘novel enrichment material’, and 8) ‘tranquilisers (e.g. azaperone)’. Farm information was collected regarding location, quality assurance scheme membership, farm size (number of pigs), housing system (indoor/outdoor/combined), average group size, and at what stages of production pigs are mixed. Personal demographic information was collected regarding gender and years of experience working with pigs. The full survey is available in supplementary materials ‘S1 Survey’.

The full study was piloted with two farmers and four researchers in order to test understanding of the choice contexts, attributes and their levels, to establish the time taken to complete the survey, and to check the appropriateness and wording of the questions. The survey was amended according to the feedback received and the final version of the survey received ethical approval from the Human Ethical Review Committee at the University of Edinburgh. Informed consent was obtained from all participants.

### Data collection

A total of 114 commercial pig farmers either completed or partially completed the survey between November 2017 and January 2019. The target population was UK and Irish pig farmers who kept pigs at the growing and/or finishing stages of production, and were responsible for making financial decisions on their farm (farm owners and managers). Farmers were recruited at nine farmer discussion groups (n = 102), at the Pig and Poultry Fair 2018 (n = 3) and by one colleague employed by the Agricultural and Horticultural Development Board (AHDB) Pork while on farm visits (n = 9). Farmer discussion groups are organised, usually quarterly, in each of the pig producing regions of the UK and each attended by between 10 and 40 farmers. Discussion groups held in Ireland are often smaller (5–15 farmers) and likely to meet more frequently. The focus of the meetings is to improve technical efficiency and profitability and they are attended by farmers from across the spectrum of the industry regarding herd size and methods of production. Animal welfare topics are not usually discussed at these meetings, and farmers were unaware that they would be asked to participate in animal welfare research prior to attending. Therefore, they were not self-selected with regards to specific welfare views, which is a risk when recruiting farmers through postal or telephone surveys. However, there is a risk that these farmers were more progressive than the population in general. Farmers’ recruited while on farm visits were unaware that they would be asked to participate in animal welfare research during the visit, and thus were not self-selected based on specific welfare views. Furthermore, these farmers were unlikely to be more progressive than the population in general.

Prior to participation all farmers were presented with standardised instructions providing information on the context of the study, the definition of the four attributes, how to interpret and complete the choice sets, and the ‘cheap talk’ script. Farmers were also asked not to talk to each other whilst filling in the survey. For participants recruited at discussion groups these instructions were presented in a PowerPoint presentation, whilst those who participated elsewhere were provided the same instructions in a paper document which the experimenter read to them before completing the survey. Participants were given the opportunity to ask questions following the instructions, prior to completing the survey.

#### Socio-demographics of the final sample

In order to analyse the choice data, it was necessary for farmers to complete all 16 choice sets. Two farmers only partially completed the choice sets and were eliminated from analysis. A further two farmers were eliminated because they did not keep growing or finishing pigs, and one was eliminated due to reporting their farm as located in the USA. Furthermore, responses from farm workers (n = 16), contract farmers (n = 2), retired farmers (n = 4) and those employed in ‘other’ roles were eliminated from analysis as these individuals had no responsibility for making financial decisions on their farm. ‘Other’ roles were advisors (n = 3), a production manager (n = 1) and an employee of the pesticide industry (n = 1).

Of the final 82 participants, 72% were farm owners and 28% were managers, and they had on average 29.7 years of experience working with pigs (std 13.5, range 4–60 yr). The majority were male (79.3% male; 12.2% female; 8.5% undisclosed), and this is consistent with the majority of agricultural workers being male in the UK and Republic of Ireland [54]. Respondents were based in England (69.5%) and the Republic of Ireland (30.5%). Although the majority of the UK pig industry is based in England (>80%), a minority is based elsewhere in Scotland, Northern Ireland and Wales [55]. Therefore the UK sample cannot be considered fully representative of the UK pig industry. All of the Irish farmers (n = 25) were assured by Bord Bia. The majority of English farmers were assured by Red Tractor (78.9%), followed by Assured British Pigs (12.3%), RSPCA (7%), and Genesis Quality (1.8%). A further 12.3% of English farmers reported having no quality assurance scheme, whilst 3.5% indicated that they were assured by another scheme (ticked ‘other’) and one farmer specified that they were assured by Marks & Spencer. Some farms were assured by more than one scheme. The proportion of farmers involved in Quality Assurance schemes in the current study is consistent with the industry whereby the majority of farmers are assured by basic schemes, such as Bord Bia and Red Tractor, which meet the minimum requirements of national legislation; but a minority meet higher than minimum standards (e.g. RSPCA)[9, 56–59]. See Table 3 for information on farm size. The majority of growing and finishing pigs were housed indoors (growers: indoors 85.4%, outdoors 0%, combined 2.4%, unspecified 12.2%; finishers: indoors 81.7%, outdoors 0%, combined 2.4%, unspecified 15.9%). Seventy-two farmers reported their average group size for growing pigs, and on average they kept pigs in groups of 47 (std 31.6, range 14–200). Sixty-nine farmers reported average group size for finishers, and they kept pigs in groups of 38 on average (std 45.3, range 12–240). Therefore the majority of pigs were housed indoors and assured by basic quality assurance schemes; furthermore farm size and structure varied widely. This is generally consistent with the structure of the industry in the UK and Republic of Ireland [55, 60].

**Table 3:** Mean, standard deviation (std), minimum and maximum number of pigs kept at each stage of production (number of farmers to answer).

Stage of production (n)	Mean	Std	Minimum	Maximum
Weaners (64)	1904	2170.6	15	10000
Growers (52)	1647	2049.4	15	10000
Finishers (62)	2548	2647.5	0	12000
Sows (63)	598	520.6	50	2184

### Discrete choice experiment modelling

All the estimations were conducted using RStudio software (Package “gmnl”). Utility refers to the net benefit from taking an action. In this choice experiment, the farmer assigned some utility to each management intervention in the choice set, and chose the one with the greatest utility. According to Lancaster (1966), any product, or in this case management intervention, is a bundle of attributes, and utilities are derived from the bundle of attributes rather than from the intervention as a whole [61]. Following this concept, what a farmer derives from an intervention is assumed to be equal to the sum of the marginal utilities for each of its attributes. Consequently, individual *i*’s assigned utility (*U*_*ijt*_) which is specific for each *j*^*th*^ intervention alternative a *t*^*th*^ choice occasion takes the form:

$$U_{ijt}=V_{ijt}+\varepsilon_{ijt}$$ (1)

*V*_*ijt*_ captures the deterministic component (which refers to the utility that the researcher captures) and *ε*_*ijt*_ the random component (which refers to all of the things that affect utility that have not been included in *V*_*ijt*_). *ε*_*ijt*_ is assumed to be independent and identically distributed for all options in each choice set. Assuming that the deterministic component is linear-in-parameter, the Eq (1) can be written as:

$$U_{ijt}={\beta X}_{ijt}+\varepsilon_{ijt}$$ (2)

Where *β* denotes the *K*×1 vector of unknown marginal utilities that are associated with the product attributes *X*_*ijt*_. In this study, *X*_*ijt*_ represents the following attributes “Installation cost” (IC), “Running cost” (RC), “Reduction in lesions” (RL) and “Improvement in growth rate” (IGR) which were coded as continuous variables using their original values. A fifth variable “None” (NONE) was also considered to estimate respondents’ preferences for the status-quo option.

The conditional logit (CL) model [62] is the most commonly used statistical model for analyzing discrete choice data. However, it assumes that respondents’ preferences are homogeneous and that alternatives included in any choice set are treated by respondents as independent. Furthermore, it assumes that all choices are independent even if they are made by the same individual and, therefore, likely exhibit some degree of correlation (i.e. in this case each farmer made 16 choices). These assumptions were found to be unrealistic and were not generally met by [63]. Revelt and Train (1998) proposed the Random Parameter Logit (RPL) model, which is less restrictive and relaxes these assumptions [64]. In the RPL model, heterogeneity of the population is taken into account as the parameters are assumed to vary from one individual to another, as is reflected in the individual-specific coefficient *β*_*i*_. The probability that individual *i* chooses an alternative *j* from a particular choice set *J* at a choice occasion *t* is specified as:

$$L_{nit}{(\beta }_{n})=\frac{\exp\left(\beta_{n}^{\prime }X_{nit}\right)}{\sum_{j=1}^{J}\exp\left(\beta_{n}^{\prime }X_{njt}\right)}$$ (3)

This probability for individual *i* is conditional on the direction and magnitude of *β*_*i*_. Since each farmer was shown a sequence of 16 choice sets, we had multiple observations for the same individual (‘panel data’). We took account of this panel dimension by computing one probability for each individual, and this was the probability included in the log-likelihood function. The choice probability of the observed sequence of choices (S) is given by:

$$S_{n}{(\beta }_{n})=\prod_{t=1}^{T}L_{ni(n,t)t}{(\beta }_{n})$$ (4)

where i(n,t) is the alternative chosen by individual n on choice occasion t.

The unconditional choice probability is the expected value of the logit probability integrated over all possible values of *β* and weighted by the direction and magnitude of *β*:

$$P_{n}(\Omega )=\int_{\beta }S_{n}(\beta )f(\beta |\Omega )d\beta$$ (5)

The expression in (5) does not have a closed form solution; therefore it is approximated through simulation methods. In particular, R draws of *β*_*ir*_ are taken from the distribution *f*(*β*|Ω). For each draw, the choice probability is calculated. Then the resulting probabilities from the R draws are averaged. We used Modified Latin Hypercube Sampling draws with 1200 simulations. The simulated log-likelihood (SLL) for all respondents, which is estimated via maximum likelihood procedures, is calculated as:

$$SLL=\sum_{n=1}^{N}ln(\frac{1}{R}\sum_{r=1}^{R}S_{n}(\beta^{r}))$$ (6)

In this study, all the parameters were assumed to be normally distributed. The results from the estimation of the RPL model showed that respondents’ preferences were highly heterogeneous. While the RPL model controls and accounts for heterogeneity, it does not explain the source of heterogeneity. Greene and Hensher (2003) proposed a latent class model (LCM) for discrete choice analysis that is less flexible than the RPL (i.e. it assumes that the distribution of respondents’ attitudes is discrete and not continuous) but it helps identify the sources of the heterogeneity. This is achieved by assuming that respondents belong to different classes, and that their attitudes are homogeneous within each class but are heterogeneous across classes. Therefore, the LCM for discrete choice analysis allows the class that a respondent belongs to be related to their characteristics and their observed behaviour (i.e. their choices of preferred combinations of attributes). In the case of LCM for discrete choice analysis, the log likelihood for all respondents is:

$$lnL=\sum_{i=1}^{N}ln[\sum_{q=1}^{Q}H_{iq}(\prod_{t=1}^{T_{i}}P_{it|q}(j))]$$ (7)

Where *H*_*iq*_ denotes the prior probability for class *q* for individual *i*. For this study, the form of the prior probability is a multinomial logit:

$$H_{iq}=\frac{exp(z_{i}^{\prime }\theta_{q})}{\sum_{q=1}^{Q}exp(z_{i}^{\prime }\theta_{q})},\;q=1,\ldots ,Q,\theta_{Q}=0$$ (8)

Where *z*_*i*_ denotes a set of observable characteristics which enter the model for class membership.

*P*_*it*|*q*_ is the choice probability that individual *i*, conditional to belonging to class q (q = 1, …, Q), chooses alternative j from a particular choice set J, comprised of j alternatives, in a particular choice occasion t, and is represented as:

$$P_{it|q}(j)=\frac{exp(x_{it,j}^{\prime }\beta_{q})}{\sum_{j=1}^{J}exp(x_{it,j}^{\prime }\beta_{q})}$$ (9)

*β*_*q*_,*θ*_*q*_ are the parameters to be estimated.

To determine the number of classes, the Consistent Akaike Information Criterion (CAIC), and the Bayesian Information Criterion (BIC) were used. Their computation and values corresponding to LCM with 2 to 5 classes are displayed in Table 4. In the following results section we report the results from the estimation of LCM with 3 classes because this model resulted in the lowest CAIC and BIC values (Table 4). CAIC was employed instead of Akaike Information Criterion (AIC) because, similar to BIC, CAIC punishes the sample size in addition to the number of parameters.

**Table 4:** Information on the latent class model (LCM) model and a random parameters logit (RPL) model. The model with the lowest CAIC and BIC values is the best fit.

Number of Classes	Log likelihood at convergence (LL)	Number of parameters (P)	Number of observations (N)	CAIC	BIC
2	-797.491	17	57	1680.714	1663.714
**3**	**-713.450**	**29**	**57**	**1573.148**	**1544.148**
4	-700.589	41	57	1607.943	1566.943
5	-677.566	53	57	1622.414	1569.414
RPL model	-1066.600	10	82	2187.387	2177.387

CAIC (Consistent Akaike Information Criterion) is calculated using: -2 * LL + (ln(N) + 1) * P

BIC (Bayesian Information Criterion) is calculated using: -2 * LL + ln(N) * P

Choice data are often used to estimate the trade-offs that respondents make between attributes, or their marginal rate of substitution. When the cost is included as the denominator in the trade-off calculations, marginal willingness to pay (WTP) can be estimated. WTP is commonly expressed as the negative ratio of the non-price attribute coefficient (e.g. reduction in lesions and improvement in growth rate) to the price (cost) coefficient (e.g. installation cost, running cost):

$${WTP}_{non-price\;attribute}=-\frac{\beta_{non\;price\;attribute}}{\beta_{price}}$$ (10)

In this study, the attributes were all coded as continuous; therefore, the calculated value represents respondents’ WTP for a one unit (1%) increase of the continuous attribute. For example, respondents’ WTP for a reduction in lesions with respect to installation cost is calculated as (-1)*(estimated coefficient for reduction in lesions/estimated coefficient for installation cost). The obtained value represents the maximum amount of installation cost that a farmer is willing to pay (or invest) to decrease lesions by 1%.

After classifying respondents in 3 classes, we characterized each class using information on respondents’ attitudes and socio-demographic characteristics. The explicative variables used in the classes’ characterization are described in Table 5. Those variables were regressed against respondent’s probability to belong to each of the three classes, which is an output of the latent class model. Since the dependent variable is in the form of a probability, we estimated a Beta regression model for each segment.

**Table 5:** Description of the variables used in the Beta regression.

Variables	Description
Mixing	Coded as 1 if the respondent mixed their pigs at the growing and / or finishing stages of production, and 0 if they did not mix pigs at either of these stages.
Small_size	Coded as 1 if the respondent owned and/or managed a small-size farm (lower quartile of dataset; 1475 or fewer growing / finishing pigs); 0 otherwise.
Medium_size	Coded as 1 if respondent owned and/or managed a medium-size farm (interquartile range of dataset; number of growing / finishing pigs between 1475–4999); 0 otherwise.
Large_size	Coded as 1 if respondent owned and/or managed a large-size farm (upper quartile of dataset; number of growing / finishing pigs 5000 or more); 0 otherwise.
Low_experience	Coded as 1 if respondent had little experience working with pigs (lower quartile of dataset; 20 or fewer years’ experience); 0 otherwise.
Medium_experience	Coded as 1 if respondent had medium experience working with pigs (interquartile range of dataset, between 21–39 years); 0 otherwise.
High_experience	Coded as 1 if respondent had much experience working with pigs (upper quartile of dataset; greater than 40 years); 0 otherwise.
Location	Coded as 1 if respondent lived in England; 0 otherwise.
Minimise_agg	Coded as 1 if respondent agreed or strongly agreed with the statement “When mixing unfamiliar pigs, minimizing aggression is important to me”; 0 otherwise.
Problem_farm	Coded as 1 if respondent agreed or strongly agreed with the statement “Mixing aggression is a problem on my farm”; 0 otherwise.
Poss_control	Coded as 1 if respondent agreed or strongly agreed with the statement “It is possible to control aggression at mixing”; 0 otherwise.
UK_standards	Coded as 1 if respondent agreed or strongly agreed with the statement “UK standards for pig welfare are sufficiently strict”; 0 otherwise.
Welfare_important	Coded as 1 if respondent agreed or strongly agreed with the statement “The welfare of my animals is important to me”; 0 otherwise.

The variables “Small_size” and “Medium_experience” and two attitudinal statements (“I avoid mixing unfamiliar pigs wherever possible” and “the welfare of my animals is good”) were not included in the analysis to avoid the problem of multi-collinearity.

## Results

### Attitudes and current practice regarding aggression

The majority of respondents reported that they avoid mixing unfamiliar pigs wherever possible (Table 6). Nevertheless only 6.4% of farmers reported never mixing unfamiliar pigs during production; 26.9% of farmers regularly mixed once during each production cycle, 38.5% mixed twice, 19.2% mixed three times, 7.7% mixed four times and one farmer mixed five times. Fattening pigs intended for slaughter were most commonly mixed at weaning (80.8%), followed by entry to the grower (25.6%) and finisher (24.4%) stages and at slaughter (16.7%). Despite the frequency of mixing, only 9.3% of farmers’ perceived aggression to be a problem on their farm (see Table 6 for results of farmers’ agreement/disagreement with each attitudinal statement). Seventy-eight percent of farmers reported that they ‘currently use’ at least one strategy to reduce aggression when they regroup unfamiliar growing/ finishing pigs on their farm. The most popular strategy was increased space allowance, followed by novel enrichment material and large social group sizes (see Table 7 for results of farmers’ use of aggression control strategies). Farmers who indicated that they employ large social groups sizes to control aggression on average kept growing pigs in groups of 48 (std = 24.8, range 16–130) and finishing pigs in groups of 38 (std = 37.5, range 12–200). Respondents were somewhat divided regarding their level of agreement with the statement ‘UK standards of pig welfare are sufficiently strict’ (see Table 6), and the majority of farmers who disagreed with this statement were based in England (n = 10), whilst two were based in Ireland.

**Table 6:** Median agreement (1 = strongly disagree, 2 = disagree, 3 = neutral, 4 = agree, 5 = strongly agree) with each attitudinal statement, and the percentage (number) of farmers to agree or strongly agree, and disagree or strongly disagree with each statement.

Attitudinal statement (n respondents)	Median response	% agree (n)	% disagree (n)
When mixing unfamiliar pigs, minimizing aggression is important to me (75)	5	68% (51)	12% (9)
Mixing aggression is a problem on my farm (75)	2	9.3% (7)	64% (48)
I avoid mixing unfamiliar pigs wherever possible (74)	5	79.7% (59)	13.5% (10)
It is possible to control aggression at mixing (73)	3	32.8% (24)	28.7% (21)
UK standards of pig welfare are sufficiently strict (68)	4	66.2% (45)	17.7% (12)
The welfare of my animals is important to me (76)	5	97.4% (64)	2.6% (2)
The welfare of my animals is good (76)	5	93.4% (71)	2.6% (2)

**Table 7:** Percentage (number) of farmers to report that they ‘currently use’, ‘used in the past’ and ‘never used’ each aggression control strategy when regrouping unfamiliar growing/ finishing pigs on their farm.

Aggression control strategy (n respondents)	Currently use (n)	Used in the past (n)	Never used (n)
Large social group sizes (65)	50.8% (33)	7.7% (5)	41.5% (27)
Increased space allowance (66)	71.2% (47)	6.1% (4)	22.7% (15)
Adding extra tryptophan to feed (65)	0% (0)	0% (0)	100% (65)
Solid visual barriers/ escape areas (66)	19.7% (13)	7.6% (5)	72.7% (48)
Mixed weight groups (65)	23.1% (15)	9.2% (6)	67.7% (44)
Mixing at night/ low light levels (64)	7.8% (5)	6.3% (4)	85.9% (55)
Novel enrichment material (68)	66.2% (45)	10.3% (7)	23.5% (16)
Tranquilisers (e.g. azaperone) (65)	4.6% (3)	12.3% (8)	83.1% (54)

### Discrete choice experiment modelling

The RPL model was estimated using all 82 participants, however, in the latent class analysis and the beta regression the number decreased to 52 due to the fact that we used respondent’s demographic and attitudinal characteristics. Missing values in any of these variables led to the respondent being omitted by the software.

The results from the RPL model (Table 8) show that preferences, as revealed by the coefficients corresponding to the main effects for each attribute, are significant and positive / negative where expected. That is, estimated installation and running cost coefficients are both negative and statistically significant, indicating that farmers prefer the installation and running costs to be cheaper. The estimated improvement in growth rate and reduction in lesions coefficients are both positive and statistically significant, indicating that farmers prefer a greater improvement in growth rate and a greater reduction in lesions. The coefficient “None” was not statistically significant indicating that farmers did not show a strong preference for either the status quo option (keeping their current farm practice) or the option to make an investment. All of the standard deviation parameters, which indicate how the valuation of the entire sample spreads around the estimated means, are significant, indicating that the preferences were heterogeneous among the sampled farmers. Since all the estimated coefficients were assumed to be normally distributed, the proportion of farmers having positive or negative valuation on each attribute can also be inferred [65]. For instance, we found that 47% of farmers preferred the status-quo option while 53% of them preferred to make an investment. Therefore, since farmers were almost equally split on both positive and negative sides of the preference scale for the status-quo alternative, the positive and negative effect cancelled each other out leading to a non-significant effect of the variable “None”. The percentages 47% and 53% were calculated using 100 X Φ(-B_k_/S_k_), where Φ is the cumulative standard normal distribution and Bk and Sk are the mean and the standard deviation, respectively, of the “None” coefficient.

**Table 8:** Estimated farmers’ preferences as indicated by the random parameter logit (RPL) model and latent class model (LCM) with 3 classes. The statistical significance associated with the standard deviation indicates whether preferences are heterogeneous among the sampled farmers.

Variables	RPL model		3 class model		
	Mean	Std	Class 1	Class 2	Class 3
None	-0.272	3.416***	15.040	0.270	-1.045***
Installation cost	-0.478***	0.306***	-18.932	-0.620***	-0.244***
Running cost	-2.391***	2.824*	-5.518	-2.877***	-0.968**
Reduction in lesions	0.028***	0.023***	-0.701	0.013	0.026***
Improvement in growth rate	0.367***	0.266***	6.412	0.455***	0.276***
Class share	1		0.18	0.32	0.50

(***) (**) and (*) denote statistical significance at (1%), (5%) and (10%) level, respectively

#### Reduction in lesions

The results from the RPL model, displayed in Table 8, show that respondents were more likely to choose an aggression control strategy that would result in a greater reduction in lesions. The computed WTP values are displayed in Table 9 and show that respondents’ WTP for an aggression control strategy increased by £0.06 per pig place (installation costs) and £0.01 per pig produced (running cost) for each 1% reduction in lesions. However, the standard deviations are significant (Table 8), which implies that farmers’ preferences were heterogeneous. Table 9 shows that among all respondents, those forming Class 1 (18% of respondents) and Class 2 (32% of participants) were unwilling to pay anything to reduce lesions. However, those in Class 3 (50% of participants) were willing to pay £0.11 per pig place (installation cost) and £0.03 per pig produced (running cost) for each 1% reduction in lesions.

**Table 9:** Farmers’ willingness to pay (WTP) (in British pounds, £).

Variables	Random parameter logit model (RPL)	3 class model		
	Mean	Class 1	Class 2	Class 3
Installation cost per 1% reduction in lesions	0.06***	-0.04	0.02	0.11***
Running cost per 1% reduction in lesions	0.01***	-0.13	0.00	0.03**
Installation cost per 1% improvement in growth rate	0.77***	0.34	0.73***	1.13***
Running cost per 1% improvement in growth rate	0.15***	1.16	0.16***	0.29**
Class share	1	0.18	0.32	0.50

(***) (**) and (*) denote statistical significance at (1%), (5%) and (10%) level, respectively

#### Improvement in growth

Farmers were more likely to invest in an aggression control strategy that provided improvements in growth rate (Table 8). In terms of WTP, farmers were willing to pay a price premium of £0.77 per pig place (installation cost) and £0.15 per pig produced (running cost) for each 1% improvement in growth rate (Table 9). However, again the estimated standard deviations are statistically significant, suggesting that farmers’ preferences for the attribute “Improvement in growth rate” were heterogeneous. The results from the latent class analysis (Table 9) show that the members of Class 1 were unwilling to pay any installation or running costs for an improvement in growth rate. Class 2 respondents were willing to pay £0.73 per pig place (installation cost) and £0.16 per pig produced (running cost) for each 1% improvement in growth. Those in Class 3 were willing to pay £1.13 per pig place (installation cost) and £0.29 per pig produced (running cost) for each 1% improvement in growth.

#### Demographic characteristics of respondent classes

Table 10 shows the results from the Beta regression analysis illustrating the demographic and production system characteristics of the members of the three classes of respondents.

**Table 10:** Results from the Beta regression–estimated marginal effects (% change in the probability to belong to one of the three classes).

Variables	Class1	Class2	Class3
Mixing	-80.58***	89.90***	-31.02***
Medium_size	96.16***	121.26***	-101.64***
Large_size	11.72**	94.27***	-56.02***
Low_experience	12.56***	-102.98***	67.04***
High_experience	30.40***	-80.47***	46.42***
Location	-20.57***	51.73***	-29.55***
Minimise_agg	3.80	-0.48	2.69**
Problem_farm	1.56	2.74	4.19***
Poss_control	-5.02*	-1.31	-1.62
UK_standards	-5.44**	-0.03	0.07
Welfare_important	4.21	3.08	-0.19
**Log pseudo-likelihood**	**150.06**	**177.92**	**139.93**
**Number of observations**	**52**	**52**	**52**
**Wald chi square**	**3071**	**37852**	**6785**
**Probability > Chi square**	**0.000**	**0.000**	**0.000**

**Class 1:** this class includes 18% of respondents. Farmers in this class were unwilling to pay any investment or running costs for an improvement in growth rates, or a reduction in lesions (Table 9). The results presented in Table 10 show that compared with the members of the other classes, members of Class 1 were least likely to mix unfamiliar growing/finishing pigs. This class were also least likely to agree with the statements ‘it is possible to control aggression at mixing’ and ‘UK standards for pig welfare are sufficiently strict’. Members of Class 1 were likely to own or manage a farm of medium size and to lesser extent large farms. Furthermore, members of Class 1 were more likely to have high experience.

**Class 2:** this class includes 32% of respondents. The members of this class were characterized by being most likely to mix unfamiliar growing finishing pigs (Table 10). However, they did not show a significant preference for strategies which reduced lesions (Table 8) and they were unwilling to pay any installation or running costs for a reduction in lesions (Table 9). They did show a significant preference for strategies which improved growth rate (Table 8) and were willing to pay £0.73 per pig place (installation cost) and £0.16 per pig produced (running cost) for each 1% improvement in growth (Table 9). Farms in this class were most likely to be of medium size and to a lesser extent large size, when compared to the other classes. They were most likely to be located in England. Farmers in this class were more likely to have medium experience.

**Class 3:** This is the largest class, including 50% of respondents. Class 3 were unlikely to mix unfamiliar growing/finishing pigs (Table 10). Nevertheless, this class stands out by comprising of members who were most willing to invest in an aggression control strategy, and were willing to pay the most for each 1% reduction in lesions (£0.11 per pig place and £0.03 per pig produced). Furthermore, they were willing to pay for an improvement in growth (£1.13 per pig place and £0.29 per pig produced) (Table 9). Farmers belonging to Class 3 were most likely to own or manage small farms, and most likely to be located in Ireland. Compared to farmers in the other classes, those in Class 3 were most likely to have low experience. Farmers in Class 3 were most likely to agree with the statements ‘when mixing unfamiliar pigs, minimizing aggression is important to me’ and ‘mixing aggression is a problem on my farm’.

## Discussion

Improving animal welfare is likely to incur some costs and to yield some benefits and farmers must trade-off estimates of these relative costs and benefits when deciding whether to implement a change in management. It is possible that a misalignment between the focus of pig aggression research and financial constraints on farmers has resulted in impractical or unaffordable solutions. This may have contributed to the limited uptake of solutions identified by research in commercial practice [2, 17]. The 82 UK and Irish pig farmers’ that participated in this study were equally split between being willing to invest in a strategy to reduce aggression between growing/finishing pigs and being unwilling to invest. The farmers who were willing to invest were heterogeneous in their preferences and willingness to pay for animal welfare improvements (i.e. reduction in lesions) and business goals (i.e. improvement in growth rate). Farmers were unwilling to invest in aggression control strategies that reduced aggression but did not improve farm productivity.

### Attitudes and practice regarding aggression

Over 90% of the farmers mixed pigs at least once at their farm, despite the fact that 79.7% reported avoiding mixing wherever possible. Weaning was the most common time for mixing amongst the surveyed farmers. These findings are roughly consistent with two prior surveys of pig farmers [17, 18]. Pigs fight in order to establish dominance relationships when regrouped, and this threatens both farm productivity and animal welfare [2, 16]. Nevertheless, only 9.3% of farmers’ in the current study perceived mixing aggression to be a problem on their farm. This is also consistent with two prior surveys of pig farmers which found that the majority of farmers did not perceive mixing aggression to be a problem [17, 18].

Minimizing aggression at regrouping was important to 68% of farmers, and 78% reported that they ‘currently use’ at least one strategy to reduce aggression when they regroup unfamiliar growing/finishing pigs on their farm. The application of these strategies by farmers is consistent with prior research which found that the most popular strategies in practice were not necessarily consistent with those found useful in the peer-reviewed literature [2, 17, 66]. Despite most farmers implementing strategies to reduce aggression, only 32.8% believed it is possible to control aggression at mixing.

With regards to animal welfare more generally, nearly all farmers valued the welfare of their animals (97.4%) and viewed the welfare of their animals to be good (93.4%). This is consistent with prior research which found that animal welfare is an important interest to farmers due to its close association with economic factors, and for ethical and public approval reasons, yet farmers do not tend to think there is anything wrong with animal welfare in livestock husbandry [67–70]. Finally, although the majority of farmers believed that UK standards of pig welfare are sufficiently strict (66.25%) a subgroup of farmers (17.7%) did not. The majority of farmers who did not believe that UK standards were sufficiently strict were based in England and not Ireland. Therefore, this was not determined by a sub-group of Irish farmers based outside of the UK perceiving UK standards as inferior. Pig farmers generally lack the intrinsic motivation to provide animal welfare standards that exceed minimum legal requirements [7]. Therefore, it is promising that in the current study a sub-group of farmers emerged that held concerns for industry standards of welfare.

### Discrete choice experiment modelling

Results revealed that, across all respondents, farmers were more likely to choose an aggression control strategy for growing/finishing pigs that resulted in a greater improvement to growth rate, a greater reduction in lesions, a lower installation cost, and a lower running cost. Respondents’ WTP for an aggression control strategy increased by £0.06 per pig place (installation cost) and £0.01 per pig produced (running cost) for each 1% reduction in lesions; and increased by £0.77 per pig place and £0.15 per pig produced for each 1% improvement in growth rate. However, the sampled farmers were not a homogenous group. Three classes of farmer were identified, each differing in their preferences and WTP for additional measures to reduce aggression when regrouping unfamiliar growing/finishing pigs on their farm. These classes also differed on several internal factors (attitudes, perceptions and personal characteristics) and farm-specific factors (size, location) which may have contributed to this heterogeneity, and can be used to identify and target these subgroups with tailored campaigns.

Those forming Class 1 (18% of respondents) did not display preferences for either an improvement in growth rate or a reduction in lesions when choosing whether to invest in an aggression control strategy for growing/finishing pigs, and were unwilling to pay for either of these attributes. However, compared to Classes 2 and 3, members of Class 1 were least likely to mix unfamiliar pigs at the growing and finishing stages of production on their farm; therefore, strategies to reduce aggression at regrouping at these stages of production were unlikely to be useful to these farmers. Farmers in Class 1 were most likely to have high experience of working with pigs; they were least likely to believe that legislative standards for pig welfare are sufficiently strict; and were least likely to believe that it is possible to control aggression at regrouping. Therefore, a reluctance to adopt aggression control strategies by these farmers was not associated with a lack of concern for animal welfare. In fact, concerns for aggression and animal welfare may have contributed to these farmers adopting higher standards of welfare by keeping pigs in stable groups throughout production.

Members of Classes 2 and 3 were both willing to change their current farm practice and invest in an aggression control strategy for growing/finishing pigs as long as certain preconditions were met; however they differed in their preferences for animal welfare and business goals, and in their WTP for these attributes. Members of Class 2 (32% of participants) were most likely to routinely mix unfamiliar pigs at growing and/or finishing. When deciding whether to invest in an aggression control strategy they were interested in the extent to which it improved growth rates, but not the extent to which it reduced aggression. They were willing to pay £0.73 per pig place and £0.16 per pig produced for each 1% improvement in growth but were unwilling to pay any investment or running costs for a reduction in aggression itself. Therefore, these farmers seem to be motivated by business but not animal welfare goals. Meanwhile, farmers in Class 3 (50% of participants) were interested in the extent to which the strategy improved growth rates, and the extent to which it reduced aggression. They were willing to pay £1.13 per pig place (investment cost) and £0.29 per pig produced (running cost) for each 1% improvement in growth rate; and £0.11 per pig place and £0.03 per pig produced for each 1% reduction in lesions, and were therefore willing to pay the most for an aggression control strategy. Farmers in Class 3 were also most likely to perceive aggression as a problem on their farm, and most likely to believe that it is important to minimise aggression at regrouping. This is consistent with prior research which found that farmers who perceived aggression to be a problem on their farm were more willing to implement aggression control strategies in the future [17], and together these studies emphasise the importance of farmers’ recognising a welfare problem where one exists.

A wide variety of aggression control strategies have been proposed by research [2, 26], and implementation of these management changes in practice may require initial investment costs (which mainly affect fixed costs), running costs (reflected in variable costs), or a combination of both. Farmers in both Classes 2 and 3 were willing to pay both investment and running costs for attributes that they were interested in. This flexibility means that a wide variety of aggression control strategies could potentially be applied in practice as farmers are not limited to, for example, strategies which only affect variable costs. However, it is currently unknown whether any existing strategies meet the financial constraints set by the farmers’ expressed WTP. Therefore, an important next step for future research is to conduct cost-benefit analyses of aggression control strategies in order to assess whether or not any existing strategies are economically viable based on compatibility with these constraints. If any of the previously identified strategies do meet these constraints, a second important step for bridging the gap between research and practice is to promote these strategies in the industry via campaigns tailored according to farmers’ preferences. For example, campaigns targeting farmers in Class 2 should emphasise the impact that specific aggression control strategies have on farm productivity, whilst campaigns targeting farmers in Class 3 should emphasise the consequences for both animal welfare and farm productivity. The most efficient way of identifying and targeting farmers in the three classes is through their socio-demographic characteristics. Specifically, farmers in Classes 1 and 2 were likely to own or manage farms of medium or large size, whilst those in Class 3 were likely to own or manage small farms. Furthermore, farmers in Class 2 were more likely to be located in England whilst those in Class 3 were more likely to be located in Ireland. These campaigns may be organised by researchers working on the communication of research findings into the industry. A final direction for future research is to develop new aggression control strategies which fit within the constraints identified in the current study.

### Study limitations

It is important to acknowledge the limitations of this study. First, the hypothetical nature of the survey limits extraneous validity, and real purchase behaviour remains unobserved. We cannot guarantee that farmers’ choices would be translated into actual buying behaviour. For example, the ethical purchase intentions of consumers are not always expressed in their actual buying behaviour [71, 72]. Nevertheless, we controlled for this as much as possible using a cheap talk script and opt-out reminders (see section 2.1 Experimental design). Second, EU pig farmers are under constant pressure as their profit margins are usually very low and fluctuate frequently [73–75]. In times where the industry is struggling farmers will have a lower WTP but their WTP may change relatively quickly depending on industry performance. Therefore the WTP of farmers identified in the current study is likely to fluctuate over time. Third, no information on farmers’ age was collected. Prior research detected a strong, positive correlation between pig farmers’ age and number of years’ experience working with pigs [76]. Therefore, it is likely that farmers’ experience in the current study is confounded with age effects. Fourth, the sample of participants was small due to difficulties in reaching this specialised demographic, and due to the fact that the population of UK and Irish pig farmers with responsibility for making financial decisions is small. This could risk Type II errors due to a lack of statistical power. Nevertheless, the sample size reflects a large proportion of the industry, and was sufficient to detect significant effects and account for heterogeneity in responses. Finally, it was not possible under the current study design to account for economies of scale, whereby costs per pig would have been lower for larger farms due to a proportionate saving in costs gained by an increased level of production. This is important because, when making an investment in animal welfare, the costs per pig would be lower for farmers in Classes 1 and 2 compared to those in Class 3.

## Conclusions

Farmers were heterogeneous in their preferences and WTP for additional aggression control strategies to use when regrouping unfamiliar growing/finishing pigs. Overall the results suggest that farmers should not be considered a homogeneous group regarding the adoption of animal welfare innovations and that researchers should target subgroups of farmers with campaigns tailored towards their preferences and WTP. This study provided an important first step in identifying economically viable solutions based on farmers’ demands. A crucial next step is to conduct cost-benefit analyses of aggression control strategies in order to assess whether any meet the demands of farmers identified here. The approach adopted in this study could be extended to other long-standing welfare issues where known solutions are poorly adopted in practice.

## Supporting information

S1 Survey The survey completed by all participants. Includes the instructions given to all participants prior to completing the choice sets, the sixteen choice sets, and questions on attitudes, farm characteristics and socio-demographic characteristics. **Data.** The data underlying the findings of this study are available at https://osf.io/xr5vu/?view_only=9c395a241be94413acfee998c9c12988
(PDF)
