# Supplementary material for: Pig farmers’ willingness to pay for management strategies to reduce aggression between pigs
Source: PLoS One. 2019 Nov 8;14(11):e0224924. doi: 10.1371/journal.pone.0224924 (PMC6839880; doi:10.1371/journal.pone.0224924)
Supplement: S1 Survey — Includes the instructions given to all participants prior to completing the choice sets, the sixteen choice sets, and questions on attitudes, farm characteristics and socio-demographic characteristics. Data. The data underlying the findings of this study are available at https://osf.io/xr5vu/?view_only=9c395a241be94413acfee998c9c12988 (PDF) [file pone.0224924.s001.pdf]

Instructions

Introduction

I would like to invite you to participate in a study which **aims to identify practical and cost-effective ways of controlling aggression** when unfamiliar pigs are mixed. This research project is part of my PhD thesis registered at the University of Edinburgh.

If you are willing to participate, you will be asked to complete a questionnaire consisting of two parts; the first involves various **choice questions**, and the second involves some **demographic** questions. In total, completing this questionnaire should not take more than 15 minutes.

If you agree to participate, your responses will remain fully confidential; no personal information will be recorded.

Your participation in this study is completely voluntary and if you do agree to participate, you can withdraw your participation at any time.

Mixing aggression

'Mixing aggression' refers to the aggression that occurs when unfamiliar pigs are mixed into new social groups. It does not include tail biting.

Aggression control strategies

A number of methods to reduce mixing aggression have been identified by research. These methods are termed 'aggression control strategies'.

Aggression control strategies usually involve changes to farm structure, management or husbandry. As a result, they can involve **investment costs** for farmers associated with new equipment and materials. They may also have **ongoing costs** associated with replenishing materials and increased labour requirements. The possible **benefits** of the investment are a **reduction in aggression** and an **improvement in production**. However, aggression control methods differ in these characteristics.

Your choices

You will be presented with **16 different choice questions**. Please note that **the structure of each question is the same**; you will be asked to choose between **three hypothetical**

aggression control strategies. The aggression control strategies are described in terms of **four characteristics** (describing their different costs and benefits), and are **unnamed** and **theoretical**. Please choose the one strategy that you are **most likely to adopt** on your farm. If you do not like any of the aggression control strategies described, you can choose a ‘no choice’ option.

We know from previous similar studies that people often answer in one way, but behave differently when they have to actually spend their money in a real world situation. When questions do not have real financial implications, people may not really consider the impact of these extra costs. Also, it is easy to be generous when you do not really have to pay for it.

So please respond to each of the following 16 questions **exactly as you would** if you were going to choose an aggression control strategy for **your farm**, and had to pay for your choice.

The following table describes the four characteristics. In the choice questions, you will see different combinations of the characteristic levels.

**Characteristics**	**Characteristic levels**
**One-off installation cost per pig place (£)**<br><br>*This refers to a one-off investment cost for installation of equipment or materials. This cost will be incurred only once.*	**£1.22per pig place**
	**£3.62per pig place**
	**£6.12per pig place**
	**£9.12per pig place**
**Running cost per pig lifetime (£)**<br><br>*This refers to an ongoing cost for the running and management of the investment. This cost will be incurred for every pig produced.*	**£0.12per pig**
	**£0.24per pig**
	**£0.34per pig**
	**£0.46per pig**
**Reduction in lesions (%)**<br><br>*This refers to the reduction in lesions in the week following mixing.*	**10% reduction**
	**20% reduction**
	**30% reduction**
	**40% reduction**
**Improvement in growth rate (%)**<br><br>*This refers to the improvement in growth rate in the week following mixing. After one week the benefit is likely to disappear.*	**0%**
	**2%**
	**5%**
	**8%**

Your choices

Please imagine that you are mixing unfamiliar pigs on your farm.

In the following 16 choice questions, carefully evaluate the three aggression control strategies and **indicate which one you would choose to use on your farm**. If you do not like any of the options, please tick the ‘no choice’ option.

PART 1. YOUR CHOICES

CONSENT

Please tick here to indicate that you understand the information given and consent to participate ☐

WHICH OF THE FOLLOWING BEST DESCRIBES YOU? *Please*			
	Farm owner		*Farm worker*
	Farm manager		*Retired*
	Contract farmer (e.g. bed & breakfast)		*Other. Please specify:*

Please remember that if you would not adopt any of the first three aggression control strategies on your farm, you should choose the 'STATUS QUO' option.

Choice set 1				
CHARACTERISTICS	UNNAMED STRATEGY 1	UNNAMED STRATEGY 2	UNNAMED STRATEGY 3	STATUS QUO
One-off installation cost per pig place (£)	£6.12	£9.12	£1.22	£0.00
Running cost per pig lifetime (£)	£0.34	£0.46	£0.12	£0.00
Reduction in lesions (%) during 7 days after mixing	10%	20%	30%	0%
Improvement in growth rate (%) during 7 days after mixing	5%	8%	0%	0%
	☐	☐	☐	☐
Please mark the option you are most likely to adopt				

Please remember that if you would not adopt any of the first three aggression control strategies on your farm, you should choose the 'STATUS QUO' option.

Choice set 2				
CHARACTERISTICS	UNNAMED STRATEGY 1	UNNAMED STRATEGY 2	UNNAMED STRATEGY 3	STATUS QUO
One-off installation cost per pig place (£)	£9.12	£1.22	£3.62	£0.00
Running cost per pig lifetime (£)	£0.46	£0.12	£0.24	£0.00
Reduction in lesions (%) during 7 days after mixing	10%	20%	30%	0%
Improvement in growth rate (%) during 7 days after mixing	8%	0%	2%	0%
	☐	☐	☐	☐
Please mark the option you are most likely to adopt				

Please remember that if you would not adopt any of the first three aggression control strategies on your farm, you should choose the 'STATUS QUO' option

Choice set 3				
CHARACTERISTICS	UNNAMED STRATEGY 1	UNNAMED STRATEGY 2	UNNAMED STRATEGY 3	STATUS QUO
One-off installation cost per pig place (£)	£3.62	£6.12	£9.12	£0.00
Running cost per pig lifetime (£)	£0.24	£0.34	£0.46	£0.00
Reduction in lesions (%) during 7 days after mixing	10%	20%	30%	0%
Improvement in growth rate (%) during 7 days after mixing	2%	5%	8%	0%
	☐	☐	☐	☐
Please mark the option you are most likely to adopt				

Please remember that if you would not adopt any of the first three aggression control strategies on your farm, you should choose the 'STATUS QUO' option.

Choice set 4				
CHARACTERISTICS	UNNAMED STRATEGY 1	UNNAMED STRATEGY 2	UNNAMED STRATEGY 3	STATUS QUO
One-off installation cost per pig place (£)	£1.22	£3.62	£6.12	£0.00
Running cost per pig lifetime (£)	£0.12	£0.24	£0.34	£0.00
Reduction in lesions (%) during 7 days after mixing	10%	20%	30%	0%
Improvement in growth rate (%) during 7 days after mixing	0%	2%	5%	0%
	☐	☐	☐	☐

Please mark the option you are most likely to adopt

Please remember that if you would not adopt any of the first three aggression control strategies on your farm, you should choose the 'STATUS QUO' option.

Choice set 5				
CHARACTERISTICS	UNNAMED STRATEGY 1	UNNAMED STRATEGY 2	UNNAMED STRATEGY 3	STATUS QUO
One-off installation cost per pig place (£)	£3.62	£6.12	£9.12	£0.00
Running cost per pig lifetime (£)	£0.46	£0.12	£0.24	£0.00
Reduction in lesions (%) during 7 days after mixing	30%	40%	10%	0%
Improvement in growth rate (%) during 7 days after mixing	0%	2%	5%	0%
	☐	☐	☐	☐
Please mark the option you are most likely to adopt				

Please remember that if you would not adopt any of the first three aggression control strategies on your farm, you should choose the 'STATUS QUO' option.

Choice set 6				
CHARACTERISTICS	UNNAMED STRATEGY 1	UNNAMED STRATEGY 2	UNNAMED STRATEGY 3	STATUS QUO
One-off installation cost per pig place (£)	£9.12	£1.22	£3.62	£0.00
Running cost per pig lifetime (£)	£0.34	£0.46	£0.12	£0.00
Reduction in lesions (%) during 7 days after mixing	20%	30%	40%	0%
Improvement in growth rate (%) during 7 days after mixing	0%	2%	5%	0%
	☐	☐	☐	☐
Please mark the option you are most likely to adopt				

Please remember that if you would not adopt any of the first three aggression control strategies on your farm, you should choose the 'STATUS QUO' option.

Choice set 7				
CHARACTERISTICS	UNNAMED STRATEGY 1	UNNAMED STRATEGY 2	UNNAMED STRATEGY 3	STATUS QUO
One-off installation cost per pig place (£)	£6.12	£9.12	£1.22	£0.00
Running cost per pig lifetime (£)	£0.24	£0.34	£0.46	£0.00
Reduction in lesions (%) during 7 days after mixing	40%	10%	20%	0%
Improvement in growth rate (%) during 7 days after mixing	0%	2%	5%	0%
	☐	☐	☐	☐
Please mark the option you are most likely to adopt				

Please remember that if you would not adopt any of the first three aggression control strategies on your farm, you should choose the 'STATUS QUO' option.

Choice set 8				
CHARACTERISTICS	UNNAMED STRATEGY 1	UNNAMED STRATEGY 2	UNNAMED STRATEGY 3	STATUS QUO
One-off installation cost per pig place (£)	£1.22	£3.62	£6.12	£0.00
Running cost per pig lifetime (£)	£0.46	£0.12	£0.24	£0.00
Reduction in lesions (%) during 7 days after mixing	40%	10%	20%	0%
Improvement in growth rate (%) during 7 days after mixing	5%	8%	0%	0%
	☐	☐	☐	☐
Please mark the option you are most likely to adopt				

Please remember that if you would not adopt any of the first three aggression control strategies on your farm, you should choose the 'STATUS QUO' option.

Choice set 9				
CHARACTERISTICS	UNNAMED STRATEGY 1	UNNAMED STRATEGY 2	UNNAMED STRATEGY 3	STATUS QUO
One-off installation cost per pig place (£)	£9.12	£1.22	£3.62	£0.00
Running cost per pig lifetime (£)	£0.12	£0.24	£0.34	£0.00
Reduction in lesions (%) during 7 days after mixing	40%	10%	20%	0%
Improvement in growth rate (%) during 7 days after mixing	2%	5%	8%	0%
	☐	☐	☐	☐
Please mark the option you are most likely to adopt				

Please remember that if you would not adopt any of the first three aggression control strategies on your farm, you should choose the 'STATUS QUO' option.

Choice set 10				
CHARACTERISTICS	UNNAMED STRATEGY 1	UNNAMED STRATEGY 2	UNNAMED STRATEGY 3	STATUS QUO
One-off installation cost per pig place (£)	£1.22	£3.62	£6.12	£0.00
Running cost per pig lifetime (£)	£0.34	£0.46	£0.12	£0.00
Reduction in lesions (%) during 7 days after mixing	30%	40%	10%	0%
Improvement in growth rate (%) during 7 days after mixing	2%	5%	8%	0%
	☐	☐	☐	☐

Please mark the option you are most likely to adopt

Please remember that if you would not adopt any of the first three aggression control strategies on your farm, you should choose the 'STATUS QUO' option.

Choice set 11				
CHARACTERISTICS	UNNAMED STRATEGY 1	UNNAMED STRATEGY 2	UNNAMED STRATEGY 3	STATUS QUO
One-off installation cost per pig place (£)	£3.62	£6.12	£9.12	£0.00
Running cost per pig lifetime (£)	£0.34	£0.46	£0.12	£0.00
Reduction in lesions (%) during 7 days after mixing	40%	10%	20%	0%
Improvement in growth rate (%) during 7 days after mixing	8%	0%	2%	0%
	☐	☐	☐	☐
Please mark the option you are most likely to adopt				

Please remember that if you would not adopt any of the first three aggression control strategies on your farm, you should choose the 'STATUS QUO' option.

Choice set 12				
CHARACTERISTICS	UNNAMED STRATEGY 1	UNNAMED STRATEGY 2	UNNAMED STRATEGY 3	STATUS QUO
One-off installation cost per pig place (£)	£3.62	£6.12	£9.12	£0.00
Running cost per pig lifetime (£)	£0.12	£0.24	£0.34	£0.00
Reduction in lesions (%) during 7 days after mixing	20%	30%	40%	0%
Improvement in growth rate (%) during 7 days after mixing	5%	8%	0%	0%
	☐	☐	☐	☐
Please mark the option you are most likely to adopt				

Please remember that if you would not adopt any of the first three aggression control strategies on your farm, you should choose the 'STATUS QUO' option.

Choice set 13				
CHARACTERISTICS	UNNAMED STRATEGY 1	UNNAMED STRATEGY 2	UNNAMED STRATEGY 3	STATUS QUO
One-off installation cost per pig place (£)	£6.12	£9.12	£1.22	£0.00
Running cost per pig lifetime (£)	£0.12	£0.24	£0.34	£0.00
Reduction in lesions (%) during 7 days after mixing	30%	40%	10%	0%
Improvement in growth rate (%) during 7 days after mixing	8%	0%	2%	0%
	☐	☐	☐	☐
Please mark the option you are most likely to adopt				

Please remember that if you would not adopt any of the first three aggression control strategies on your farm, you should choose the 'STATUS QUO' option.

Choice set 14				
CHARACTERISTICS	UNNAMED STRATEGY 1	UNNAMED STRATEGY 2	UNNAMED STRATEGY 3	STATUS QUO
One-off installation cost per pig place (£)	£6.12	£9.12	£1.22	£0.00
Running cost per pig lifetime (£)	£0.46	£0.12	£0.24	£0.00
Reduction in lesions (%) during 7 days after mixing	20%	30%	40%	0%
Improvement in growth rate (%) during 7 days after mixing	2%	5%	8%	0%
	☐	☐	☐	☐
Please mark the option you are most likely to adopt				

Please remember that if you would not adopt any of the first three aggression control strategies on your farm, you should choose the 'STATUS QUO' option.

Choice set 15				
CHARACTERISTICS	UNNAMED STRATEGY 1	UNNAMED STRATEGY 2	UNNAMED STRATEGY 3	STATUS QUO
One-off installation cost per pig place (£)	£9.12	£1.22	£3.62	£0.00
Running cost per pig lifetime (£)	£0.24	£0.34	£0.46	£0.00
Reduction in lesions (%) during 7 days after mixing	30%	40%	10%	0%
Improvement in growth rate (%) during 7 days after mixing	5%	8%	0%	0%
	☐	☐	☐	☐
Please mark the option you are most likely to adopt				

Please remember that if you would not adopt any of the first three aggression control strategies on your farm, you should choose the 'STATUS QUO' option.

Choice set 16				
CHARACTERISTICS	UNNAMED STRATEGY 1	UNNAMED STRATEGY 2	UNNAMED STRATEGY 3	STATUS QUO
One-off installation cost per pig place (£)	£1.22	£3.62	£6.12	£0.00
Running cost per pig lifetime (£)	£0.24	£0.34	£0.46	£0.00
Reduction in lesions (%) during 7 days after mixing	20%	30%	40%	0%
Improvement in growth rate (%) during 7 days after mixing	8%	0%	2%	0%
	☐	☐	☐	☐
Please mark the option you are most likely to adopt				

PART 2. QUESTIONNAIRE

<u>IF YOU ANSWERED 'STATUS QUO' TO ALL CHOICE SETS</u> **please let us know why by ticking all relevant boxes:**	
	I would not adopt any of the aggression control strategies on my farm
	I already use aggression control strategies on my farm
	I don't mix unfamiliar growers/finishers
	Didn't read the other options
	Wanted to get finished quickly

Other, Please state:……………………………………………………………………………………………

**Please indicate your level of agreement/disagreement with the following statements.**	*Strongly disagree*				*Strongly agree*
When mixing unfamiliar pigs, minimizing aggression is important to me	1	2	3	4	5
Mixing aggression is a problem on my farm	1	2	3	4	5
I avoid mixing unfamiliar pigs wherever possible	1	2	3	4	5
It is possible to control aggression at mixing	1	2	3	4	5
UK standards for pig welfare are sufficiently strict	1	2	3	4	5
The welfare of my animals is important to me	1	2	3	4	5
The welfare of my animals is good	1	2	3	4	5

When do you routinely mix unfamiliar pigs on your farm? *Please tick all that apply*

	Never		*At finisher*		*Other, please specify:*
	At weaning		*Before slaughter*		…………………………………………………………
	At grower		*Sows*		

Roughly how many of the following do you have on your farm at any time?

**Weaners:**		**Finishers:**	
**Growers:**		**Sows:**	

**What is the average group size of growers on your farm?**	

**What is the average group size of finishers on your farm?**	

How are growing and finishing pigs housed on your farm?

**Growers:**	*Indoor*	*Outdoor*	*Combined*
**Finishers:**	*Indoor*	*Outdoor*	*Combined*

**When mixing growing/finishing pigs, please indicate your use of the following aggression control strategies.** *Please circle your answer*			
Large social group sizes:	*Currently use*	*Used in the past*	*Never used*
Increased space allowance:	*Currently use*	*Used in the past*	*Never used*
Adding extra tryptophan to feed:	*Currently use*	*Used in the past*	*Never used*
Solid visual barriers/ escape areas:	*Currently use*	*Used in the past*	*Never used*
Mixed weight groups:	*Currently use*	*Used in the past*	*Never used*
Mixing at night/ low light levels:	*Currently use*	*Used in the past*	*Never used*
Novel enrichment material:	*Currently use*	*Used in the past*	*Never used*
Tranquilisers (e.g. azaperone):	*Currently use*	*Used in the past*	*Never used*
Avoidance of mixing:	*Currently use*	*Used in the past*	*Never used*

**Are you a member of a Quality Assurance Scheme?** *Please tick all that apply*					
	No scheme		*Scottish SPC accreditation*		*Genesis Quality*
	RSPCA Assured		*Quality Meat Scotland*		*Red Tractor*
	Soil Association		*Assured British Pigs*		*Other, please specify:*

ABOUT YOU:

**Gender**:	*Male*	*Female*	*Prefer not to say*

**Roughly how many years have you worked with pigs?**	

**Location:**			
	England		*Northern Ireland*
	Scotland		*Republic of Ireland*
	Wales		*Other. Please specify:*

Thank you for taking the time to complete this survey.

Your insight and information are very valuable to us in directing our research towards demand driven solutions to reduce aggression between pigs.
